# Multidisciplinary approach to COVID-19 and cancer: consensus from scientific societies in Argentina

**DOI:** 10.3332/ecancer.2020.1044

**Published:** 2020-05-13

**Authors:** Julia Ismael, Federico Losco, Sergio Quildrian, Pablo Sanchez, Isabel Pincemin, Jose Lastiri, Santiago Bella, Alejandro Chinellato, Guillermo Dellamea, Alejandro Ahualli, Silvana Rompato, Julio Velez, Rafael Escobar, Ariel Zwenger, Cristina Rosales, Claudia Bagnes, Jorge Puyol, Dario Niewiadomski, Edgardo Smecuol, Fabio Nachman, Eduardo Gonzalez, Gustavo Ferraris, Juan Ramos Suppicich, Paola Price, Luis Medina, Juan O’Connor

**Affiliations:** 1 Asociación Argentina de Cirugía, Marcelo T de Alvear 2415, 1122AAM, Buenos Aires, Argentina; 2 Asociación Argentina de Medicina y Cuidados Paliativos, Av Rivadavia 1255 of 309 C1033AAC, Buenos Aires, Argentina; 3 Asociación Argentina de Oncología Clinica, Av Federico Lacroze 2252, C1426 CPU, Buenos Aires, Argentina; 4 Asociación de Oncología de Rosario, Sta Fe 1798, Rosario S2000AUB, Argentina; 5 Asociación de Oncología del Chaco, Av Avalos 468H3500BZR, Chaco, Argentina; 6 Asociación de Oncólogos de Cordoba, Ovidio Lagos 226, X5004 ACF, Cordoba, Argentina; 7 Asociación Formoseña de Oncología Clinica, Padre Patiño 260, P3600 KWE, Argentina; 8 Asociación Oncología Clinica de Corrientes, Necochea 1050 C3400, Corrientes, Argentina; 9 Endoscopistas Digestivos de Buenos Aires, Dr Tomás Manuel de Anchorena 1357, 1123, Caba, Argentina; 10 Fundación Oncológica de la Patagonia, Av Francisco de Viedma 1202, R8500AYY, Río Negro, Argentina; 11 Red de Oncología de CABA, Avenida Patricias Argentinas 750, C1405BWU, Argentina; 12 Sociedad Argentina de Cancerología, Av Santa Fe 1171 C1059ABF, Argentina; 13 Sociedad Argentina de Gastroenterología, Marcelo T de Alvear 1381 Piso 9, C1058AAU, Buenos Aires, Argentina; 14 Sociedad Argentina de Mastología, Marcelo Torcuato de Alvear 1252, C1058 AAT, Buenos Aires, Argentina; 15 Sociedad Argentina de Terapia Radiante, Avenida Santa Fé 1171 C1059ABF, Argentina; 16 Sociedad Argentina de Urología, De la Cárcova 3526, C1174, Buenos Aires, Argentina; 17 Sociedad de Cancerología de La Plata, 50 374, La Plata (1900), Buenos Aires, Argentina; 18 Sociedad de Oncología Clinica de Tucuman, Las Piedras 496, T4000 BRJ, Argentina

**Keywords:** COVID-19, coronavirus, recommendation, guidelines, cancer

## Abstract

**Introduction:**

The world is living through an outbreak of an acute respiratory syndrome caused by a new betacoronavirus known as coronavirus 2 (SARS CoV-2), which has been declared an international public health emergency by the World Health Organisation. Cancer patients are a very special population in this setting since they are more susceptible to viral infections than the general population. Several recommendations have been made on this issue, most of them based on expert opinion and institutional experience. It is essential to gather the evidence available for decision making.

**Objective:**

To review the evidence available in order to create a multi-institutional position from the perspective of scientific societies in Argentina involved in the management of cancer patients.

**Methodology:**

The review included two phases: 1) search and systematic revision of the medical literature; 2) consensus and revision of the document drafted by national scientific societies involved in the management and care of cancer patients using the modified Delphi method. The final results were presented at a videoconference with all the participants. Also, additional comment and recommendations were discussed. The final document was revised and approved for publication by the members of the panel.

**Results:**

The consensus panel included 18 representatives from scientific societies from Argentina who assessed the evidence and then made recommendations for the management of cancer patients in our country. International guidelines (CDC; ASCO, NCCN and ESMO) were considered as a background for analysis, as well as institutional guidelines and an open *ad hoc* survey administered to 114 healthcare professionals from the scientific societies involved in this study.

The recommendations are grouped as follows: 1) general care interventions—training of the personnel, cleaning and disinfection of the hospital premises and patient scheduling; 2) treatment decisions—patient care, surgeries, immunosuppressive therapy, radiotherapy and screening; 3) ethical considerations—optimisation of resources, end-of-life care for critically-ill patients; 4) management of hospitalised patients; and 5) wellbeing of the healthcare team.

The general recommendation arising from the study is that the management of cancer patients must adapt to the exceptional pandemic status quo without disregarding treatment or cure options. Moreover, healthcare professional accompaniment of all patients should not be neglected. All healthcare professionals must make a significant joint effort to create multidisciplinary teams to discuss the most appropriate measures for each particular situation.

**Conclusions:**

The scientific evidence available on this topic worldwide is in progress. This together with the epidemiologically shifting scenario poses unprecedented challenges in the management of cancer amidst this global pandemic. Furthermore, the key role of the healthcare structural organisation appears evident, such as the drafting of clear guidelines for all the stakeholders, adaptability to constant change and an interdisciplinary shared vision through consensus to provide adequate care to our cancer patients in the light of uncertainty and fast-paced change.

## Introduction

The world is experiencing an outbreak of acute respiratory syndrome caused by a new betacoronavirus known as coronavirus 2 (SARS-CoV-2) [1]

By 27 March, 2020, the rapid spread of the virus had resulted in 509,164 confirmed cases and 23,335 deaths. Cases have been reported in 136 countries. The World Health Organisation (WHO) has declared the new coronavirus disease (COVID-19), caused by SARS-CoV-2, is a public health emergency of international concern. In contrast to the severe acute respiratory system, coronavirus and the Middle East respiratory syndrome-related coronavirus, COVID-19 has caused more deaths from multiple organ dysfunction syndrome than from respiratory failure [2], which could be attributable to the widespread distribution of angiotensin-converting enzyme 2—the functional receptor for SARS-CoV-2—in multiple organs [3, 4].

Pre-existing medical conditions have been defined by the United States Centers for Disease Control and Prevention (CDC) [5] as increasing the risk of COVID-19 infection, including:

- People with chronic respiratory diseases: chronic obstructive pulmonary disease, congenital emphysema, bronchopulmonary dysplasia, bronchiectasis, cystic fibrosis and moderate or severe asthma.
- People with heart conditions: heart failure, high blood pressure, coronary heart disease, heart valve disease and congenital heart disease.
- Immunocompromised individuals, which includes those undergoing cancer treatment.
- People of any age with severe obesity (body mass index (BMI) ≥ 40) or certain underlying medical conditions, particularly if not well controlled—such as those with diabetes, renal insufficiency or liver disease—may also be at risk.

Patients with cancer are more susceptible to viral infections than those without, possibly because of the state of immunosuppression caused as much by the malignancy of the cancer as by the treatments, such as chemotherapy or surgery [6–9].

According to the latest estimates by the International Agency for Research on Cancer based on globally available data for the year 2018, Argentina has an incidence rate of 212 cases per 100,000 inhabitants (taking into account both sexes and all tumours except for non-melanoma skin cancer); a figure that positions it within the countries of the world with medium-high cancer incidence (range of 177 to 245.6 per 100,000 inhabitants), placing it in seventh place in Latin America. This estimate corresponds to more than 125,000 new cases of cancer in both sexes per year [10]. Thus, it is a prevalent disease in our country.

As knowledge about COVID-19 (Coronavirus disease 2019, COVID-19) increases, specific recommendations regarding cancer have advanced significantly. However, most of the documents produced internationally come from expert opinions and institutional experiences, and should be regarded with caution. It should not be forgotten that the evidence on this pathology is dynamic and is permanently under construction.

## Objective

Multi-institutional position from the perspective of the scientific societies of Argentina deals with the management of oncology patients.

## Methodology

The implementation of this inter-societal consensus was planned in two stages as follows:

### 1) Systematic literature search

A systematic literature search was carried out by two independent researchers on PubMed and Google Scholar, supplemented by manual searches on national and international scientific society pages. The search was carried out on 6 April, 2020.

#### Search strategy

PubMed terms: (‘neoplasms’ [MeSH Terms] OR ‘neoplasms’ [All Fields] OR ‘cancer’ [All Fields]) AND (‘COVID-19’[All Fields] OR ‘COVID-2019’ [All Fields] OR ‘severe acute respiratory syndrome coronavirus 2’ [Supplementary Concept] OR ‘severe acute respiratory syndrome coronavirus 2’ [All Fields] OR ‘2019-nCoV’ [All Fields] OR ‘SARS-CoV-2’ [All Fields] OR ‘2019nCoV’ [All Fields] OR ((‘Wuhan’ [All Fields] AND (‘coronavirus’ [MeSH Terms] OR ‘coronavirus’ [All Fields])) AND (2019/12[PDAT] OR 2020[PDAT]))).

Google Scholar terms: (Cancer AND (‘COVID-19’ OR ‘2019ncov’ OR ‘2019 ncov’ OR ‘novel coronavirus’ OR ‘novel corona virus’ OR covid 19 OR ‘covid 19’ OR ((coronavirus OR ‘corona virus’ OR cov OR ncov) AND (outbreak OR wuhan)) OR ((coronavirus OR ‘corona virus’) AND 2019))).

#### Search results

Priority was given to the inclusion of clinical practice guidelines, other systematic reviews, or meta-analyses.

Within the results obtained, publications containing recommendations from scientific societies that could be adapted to a national context were selected. Given the current available evidence, the source of the evidence is based on observational studies with a low number of patients and recommendations based on the consensus of experts.

Relevant guides were extracted from the National Comprehensive Cancer Network (NCCN), the CDC, the American Society of Clinical Oncology (ASCO), and the European Society for Medical Oncology (ESMO), added to recommendations from national scientific societies (see Appendix III).

### 2) Consensus development

#### Formation of the panel

The authorities from national scientific societies were convened in order to appoint a participating member. His/her function will be to represent the institutional position with a single vote when developing the questionnaire.

18 societies were invited and accepted the invitation to participate.

The list of participating societies, representative and conflicts of interest are detailed in Appendix I.

#### Research questions

Once the pertinent and relevant evidence was identified, the points or questions of controversy to be developed in relation to the proposed theme were extracted.

A list was generated following ASCO and NCCN guidelines.

The questionnaire was subsequently distributed among the members of the panel to validate the different aspects of the topic to address, and to modify or add relevant questions not covered in the initial proposal.

The final questionnaire set out in this document was eventually agreed upon (Appendix II).

Consensus methodology: The modified Delphi method was used. This is an iterative process, whereby participants must give their opinions or answers anonymously on more than one occasion, via rounds that result in opinion stability. It is characterised by feedback controlled by the coordinating group at each stage, through which a statistical response for the group is reached.

A first round of 10 questions was conducted using the Survey Monkey® tool, outlining a percentage of agreement of greater than 60%.

By decision of the elaboration group, the same survey was carried out with an open and non-nominalised format by the members of the intervening societies, with the aim of finding out the general perception of the subject in different areas of focus. There were 114 participants.

#### Results

The final results were presented in a video conference with all of the participants, where additional comments and recommendations were brought up.

The results of the open survey were also shared, for the purpose of enriching the discussion space.

In cases where the established percentage was not reached, a second vote was taken in order to come to an agreement during the video conference meeting.

A draft document was prepared, which was sent by email for the members of the panel to review and conclude, in order to arrive at a final version of the document.

## General recommendations for the management of patients diagnosed with cancer in the context of the SARS-Cov-2/COVID-19 pandemic

Recommendations from ASCO [11], NCCN [12] and ESMO [13] have charted them on six axes, which respond to these questions of healthcare practice for oncology patients, through evidence and the agreement of experts. We have supplemented this with local recommendations in the same order.

### Preparation

#### General care

What are the recommendations for the general care of cancer patients?

Points of practice may be considered to guide the clinic’s preparation and planning:

#### Preparation of staff

Clinic or hospital staff may need additional training to assess patients for possible COVID-19 / other infections [5].

The procedures for isolating potentially infected patients may need to be reviewed and updated.

Clinic staff may need additional training on the use of personal protective equipment (PPE).

It may be necessary to obtain additional PPE, as staff who do not usually use it may be required to perform tasks where it is appropriate.

Clinic or hospital staff may need additional training on how to obtain SARS-CoV-2 testing for patients according to the current testing guidelines.

#### Cleaning and disinfecting the hospital environment

The recommendation for this is to follow the inter-institutional recommendations to prevent COVID-19—version 22/03/2020 of the Argentine Society of Infectious Diseases (SADI) / Argentine Intensive Care Society (SATI) / Infection Control Nurses Association (ADECI) / Institute of Epidemiology—Dr Juan H Hara [14].

#### Patient scheduling

It may be reasonable to postpone routine follow-up visits for patients who are not undergoing active cancer treatment, or make those appointments by telemedicine.

Consider calling all scheduled patients 1 day before they visit the clinic in order to detect COVID-19 exposure/symptoms.

Routine collection of laboratory samples from the patient’s home may be considered, instead of having them come into the clinic.

Treatment planning:

1. For patients with a fever or other symptoms of infection, an in-depth evaluation must be carried out according to usual medical practice.
2. Patients with COVID-19 currently receiving treatment against cancer must follow standard clinical management plans to delay or modify cancer treatment in a patient with an active infection.

Current information suggests that patients with cancer are at a greater risk of infection and severe complications from COVID-19 than other patients. For the majority of cases where there is no known COVID-19 infection, it is likely to be more important to start or continue systematic treatment, than delay or interrupt it over concerns about possible COVID-19 infection. However, decisions should be made on an individual basis after considering the age of the patient, the general objectives of the treatment, the present oncological state of the patient and tolerance to treatment, as well as their general medical condition.

Consider if domiciliary infusion of chemotherapy medication is feasible from a medical and logistical point of view for the patient, medical staff and caregivers. In our country, legal aspects must also be taken into account.

Given that oncology patients are also at a high risk of serious complications from influenza, they should be vaccinated before starting chemotherapy (at least 2 weeks before if possible), and in cases where chemotherapy has already started, it should be done immediately before the next cycle of treatment [15].

### Treatment decisions

#### Patient care

How do you see the care of patients with cancer affected by the current COVID-19 pandemic?

**Information:** *What is the current information on cancer patient care and COVID-19?*

CDC guidelines: Patients should be informed about COVID-19 symptoms and trained in hand washing, hygiene and social distancing.

Initially, there was no formal recommendation regarding the use of masks with cancer patients [5]. In the recommendations for health workers from the Department of Health in our country, they mention the use of masks by staff who care directly for isolated patients. For example, patients with influenza, coronavirus (including COVID-19), respiratory syncytial virus, meningococcus, mumps, rubella and patients with respiratory infections walking around the hospital [16].

In an update of the guidelines, the CDC recommends the use of masks for healthy people who are going to be exposed to the public, or for patients who must attend health centres; even more so for cancer patients, due to the risk posed by their illness. Protection with masks/face coverings is complementary to maintaining distance and isolation as part of preventative measures [17]. Before symptoms are present, the usual early clinical evaluation is recommended [5]. With respect to systematic COVID-19 testing, there is no conclusive evidence.

Is it more likely for cancer patients to become infected?

Even though no conclusive increase has been established in the number of infections by COVID-19 in people with an oncological diagnosis, there is evidence that they are more at risk of developing severe forms of the illness, with a higher death rate [18].

Do they have more complications?

At the moment, only one detailed published report has been identified that compares the spread of the COVID-19 virus in the patients with and without cancer [1]. This report outlines a prospective cohort of 1,571 patients with COVID-19, 18 of which had a history of cancer. They observed that patients with a history of cancer had more severe complications. This was defined as the percentage of patients admitted to one of the intensive care units who required invasive ventilation or died, in comparison with other patients. According to correspondence from the report, Xia *et al* [2], these 18 patients represent a heterogeneous group and are not an ideal representation of the entire population of cancer patients. Another recent publication, based on cases of cancer and COVID-19 from three hospitals in Wuhan, reports outcomes from 28 patients. In these cases, 53.6% developed severe forms of the illness and 28.6% were related deaths [18].

#### Surgeries

Can surgeries be cancelled or delayed?

The CDC suggests ‘elective surgeries’ are rescheduled if possible.

The American College of Surgeons has also given a recommendation.

However, doctors and patients should discuss individual cases, evaluating the potential harm of delaying cancer related surgeries; without which they risk missing the window of opportunity for surgical treatment of the patient. In many cases, these surgeries cannot be considered as ‘elective [19].

#### Immunosuppressive therapy

Can immunosuppressive therapy be cancelled, delayed or interrupted?

Treatment plan

For patients with a fever or other symptoms of infection, a detailed evaluation should be carried out according to usual medical practice.

For patients with COVID-19 who are currently receiving treatment for cancer, consider delaying or changing the cancer treatment for patients with active infection.

The current information suggests that cancer patients are at a higher risk of infection and complications from COVID-19 than other patients. For patients with no known COVID-19 infection, in the majority of cases, it is likely to be more important to start or continue systematic cancer treatment, than delay or interrupt it over concerns about possible COVID-19 infection. However, decisions should be made on an individual basis after considering the overall objectives of treatment, the current oncological state of the patient and their tolerance to treatment, as well as their general medical condition.

At the moment, there is no direct evidence to support the change or suspension of chemotherapy or immunotherapy in cancer patients. Therefore, suspending routine anticancer or immunosuppressive therapy is not recommended. The balance of possible harm that could result from delaying or interrupting treatment versus the possible benefits of preventing or delaying COVID-19 infection is very unclear. Clinical decisions should be individual considering factors such as the risk of the illness recurring if adjuvant chemotherapy is delayed, modified or interrupted, as well as the number of adjuvant chemotherapy cycles already done and the tolerance of the patient to treatment.

However, the following practices should be considered:

1. For patients largely in remission undergoing maintenance therapy, stopping chemotherapy may be an option.
2. Some patients may change from intravenous to oral chemotherapy, which would decrease the frequency of visits to the clinic, but would require greater care from medical teams to ensure the correct prescription is met.
3. Decisions about modifying or suspending chemotherapy should consider chemotherapy advice and treatment objectives, as well as the clinical characteristics of the patient and their tolerance to treatment. For example, the risk/benefit evaluation of continuing with chemotherapy in untreated small cell lung cancer patients is different to patients being maintained with pemetrexed for non-small cell metastatic lung tumours.
4. If local transmission affects one oncology centre in particular, reasonable options may include offering a chemotherapy break for 2 weeks, organising infusion at a remote unaffected unit, or coordinating treatment with another unaffected centre.
5. Consider home infusion if it is medically and logistically feasible for the patient, medical team and caregivers. In some environments, the delay or change of adjuvant treatment may mean putting infection control at risk, compromising long-term survival.

Prophylactic growth factors that would be used in high-risk neutropenia chemotherapy regimes, as well as prophylactic antibiotics, can have potential value in maintaining the general health of the patient and make them less vulnerable to possible complications from COVID-19.

In cases where the overall benefit from adjuvant chemotherapy may be small, and where there are no immunosuppressive options available (for example, hormonal therapy in early-stage hormone-dependant breast cancer), the risk of infection from COVID-19 can be considered as an additional factor to evaluate before the different options available to the patient.

**Immunotherapy:** It is important in the context of active treatment in cancer patients. It includes the use of agents such as PD-1 inhibitors, for example, nivolumab and pembrolizumab and anti CTLA-4 therapy such as ipilimumab.

On this point, consider 3 aspects [20]:

1. It is possible that treatment restores the immune system, creating a similar picture to that developed in severe forms of COVID-19 (caused by a massive release of cytokines).
2. Consider the subtype of the tumour and the objective of the treatment to decide whether to continue or start treatment with PD-1 inhibitors (e.g. melanoma, kidney cancer).
3. Even if the alveolitis was generated with toxicity by the PD-1 inhibitors is not a frequent occurrence, it can be the cause of death in up to 35% of cases. In patients with comorbidity or a respiratory disease, it is necessary to consider the increased risk when using PD-1 inhibitors, mainly when combining PD-1 inhibitors with anti CTLA 4 therapy.

#### Radiotherapy

In summary, here are 10 recommendations [21]:

1. Only the patient should enter the centre, wearing a mask and keeping a safe distance from others.
2. First-time and post-treatment consultations should take place via video call or telephone.
3. Defer starting treatment in early-stage cases of the illness. These cases should have been previously discussed by the Tumour Committee.
4. Toxicity should be controlled when requested by the patient.
5. As many treatments as possible should be hypofractionated while following international protocol.
6. Reduce treatment time in accordance with the volume of each centre.
7. High-risk staff should work remotely.
8. Patients suspected to have COVID-19 should not be treated until diagnosed.
9. Patients who have tested positive for COVID-19 should be urgently treated in the final hourly schedule and protective measures should be taken.
10. Create working groups so that those possibly infected with the virus are not in contact with other patients.
11. Staff with any suspected symptoms must report themselves and refrain from entering the centre.

**Confirmed cases of COVID-19:** Treatment will be limited to urgent cases, such as patients with spinal cord compression, superior vena cava syndrome or bleeding identified by a specialist. In this situation, it is vital to give priority to hypofractionated treatments, especially with a single dose (8 Gy or 18 Gy in 3 Fx). For non-urgent cases, treatment will be paused depending on the individualised decision for each patient. Individual and group protection is defined accordingly by the rules established by the Ministry of Health. The last shift of each day will be dedicated to treating patients with suspected or confirmed cases of COVID-19.

Diagnoses in which it is feasible to omit starting radiotherapy treatment in breast cancer patients are: hormone receptor-positive ductal carcinoma (DCIS, RH+); patients over 65 years who are receiving hormonal treatment after carrying out an individualised evaluation of the risks and benefits of adjuvancy treatment alongside radiotherapy; DCIS in patients under 65 years who meet the low-risk criteria (clinically hidden tumour, detected by mammogram, screening or incidental findings during surgery amounting to a 3 mm with a nuclear grade 1 or 2). In these hormone receptor-positive cases, endocrine therapy needs to be completed. In cases of stage IA (cT1 N0) luminal A invasive ductal carcinoma for those over 70 years who receive hormone therapy and palliative cases with a luminal illness with an indication of hormone therapy.

**Prostate cancer:** depending on the clinical characteristics and the risk subgroup, the use of neoadjuvant androgen deprivation therapy should be evaluated. In case of low-risk or very low-risk illnesses, it is recommended to continue active surveillance. For intermediate-risk diseases, defer starting treatment during the contingency period.

For localised high-risk and very high-risk diseases with positive ganglions, it is not advisable to start treatment during the contingency period when neoadjuvant androgen deprivation therapy is the proposed method of treatment. For advanced illnesses, delay starting treatment within this period. Such illnesses are based on the STAMPEDE protocol or ablative therapies of metastatic diseases or M1.

All patients must continue androgen deprivation therapy. For patients with a biochemical recurrence and without a distant disease, it is recommended to begin hormone therapy.

Consider postponing radiotherapy in treatments with palliative intent in asymptomatic or oligosymptomatic patients, or in response to medical management.

**Brachytherapy:** palliative brachytherapy is recommended for skin tumours.

#### Screening

Should members of the community continue with recommended activities for detecting cancer (e.g., mammograms)?

To conserve resources in the health system and limit contact with patients at healthcare facilities, ASCO recommends that procedures requiring visits to clinics and centres, such as mammograms and colonoscopies, should be postponed for the time being. It is recommended that healthcare teams carefully consider the risks and benefits of continuing elective procedures, such as screening procedures, at this moment.

#### Digestive Endoscopy

Digestive endoscopy generates aerosols [21, 22]. The viral load of SARS-CoV-2 in the pharyngeal saliva is similar to that in stool, where it can linger for 2 days after respiratory sample results indicate they are negative [23]. Up to 62% of infections with SARS-CoV-2 occur during the presymptomatic phase [24]. The core recommendations of the Spanish Association of Digestive Endoscopy (SEED) during the COVID-19 pandemic consist in:

1. Limiting endoscopic procedures to urgent cases only
2. Carrying out endoscopic procedures by ensuring it is performed with an adequate level of protection [25]

Endoscopist responsibilities:

- Under the oath to do no harm that doctors have taken, the SEED reminds us that all endoscopic doctors must currently:
  - Suspend all scheduled endoscopic outpatient activity.
  - Without delay, be available to carry out urgent endoscopies.
  - Limit digestive endoscopy to vital hospital activity, such as in:
    - Patients with upper gastrointestinal bleeding who suffer from haemodynamic instability, when it is possible to carry out endoscopic treatment.
    - Patients with oesophageal impaction from foreign bodies.
    - Patients with obstructive cholangitis who require an ERCP.
    - Cancer patients who require endoscopic treatment.
    - Specific colonoscopies, where diagnostic yield is urgent.
  - Protect themselves and all personnel by properly using PPE [26, 27].

### Ethical considerations

Beyond the care of each individual patient, medical oncologists will have to face the reality of limiting care. As the pandemic advances, a point will arrive at which channelling a large number of resources for an individual patient will directly conflict with the greater social good. If a cancer patient at an advanced stage of the illness or with comorbidities, such as cardiac or pulmonary dysfunction, becomes infected with COVID-19 and requires mechanical ventilation, it’s possible that it’s a bleak prognosis. Based on a recent retrospective study from Wuhan, China, only one patient survived from the 32 who were all gravely ill with confirmed cases of COVID-19 and required mechanical ventilation [3a large number of SARS-related coronaviruses (SARSr-CoVs].

Therefore, it is imperative to have proactive discussions about end of life arrangements and palliative care with cancer patients at risk of infection by COVID-19. The proactive discussions surrounding these challenging decisions should occur among specific groups of illnesses, between specialists in medical ethics and palliative care teams.

In triage procedures, where patients with a shorter life expectancy should not be treated, palliative care teams insist that these patients should not be abandoned, but still provided with the adequate health care needed. In this regard, it is convenient to adapt spaces for patients with a shorter life expectancy. Such will avoid overburdening healthcare workers on the front line who will suffer extreme stress seeing patients pass from untreated symptoms and who will at times be unable to exercise their necessary skill and expertise.

From an ethical perspective, we can assume the following criteria:

1. **Protection from harm.** A flu pandemic and classification system can cause significant physical and emotional distress. It is our obligation to protect those who suffer from the effects of the pandemic and classification system.
2. **Proportionality.** The administration of resources in palliative care must reflect the needs of the community and high-quality palliative care must be provided to those who need it. Medication, equipment, beds and specialists must not ‘accumulate’ when they are needed by others in the community.
3. **Obligation to provide access to care.** Palliative care is a critical component of any pandemic classification plan and the professionals working within palliative care have an obligation to provide care and alleviate suffering. This must be balanced by the obligation to protect the health of oneself and that of their families.
4. **Reciprocity.** Palliative resources should be directed more to patients who have greater burden on the classification system, such as critical patients who will not receive life support treatment. These patients (and their families) run a greater risk of feeling abandoned by the healthcare system and should receive care based on the therapeutic objective for comfort by an interdisciplinary palliative care team.
5. **Equity.** We must ensure that high-quality palliative care is available for all patients who may need it, regardless of their ranking on the scale.

The Argentine Association of Medicine and Palliative Care (AAMYCP) have made the recommendations outlined in this document (see Appendix III), https://aamycp.com.ar/covid19/.

### Hospital management of oncology patients

The priority for oncology units for hospitalised patients has been to prepare for the upcoming bed and resources shortage caused by the expected increase in COVID-19 patients who require beds and acute care in intensive care units (ICU). Even though some centres have beds or facilities that are allocated for cancer care, the rapid increase in cases may require the reassignment of units, hospital wards or even entire systems for patient care during the pandemic. Shortage of blood products due to the drop in blood donors within the community calls for stricter usage guidelines both for clinics and hospitals [28]. Transfer of potentially infected patients from one centre to another requires careful logistics planning in order to reduce both public and staff exposure. Due to shortage of supply and increased demand, creative solutions must be developed to preserve PPE, including favouring hand-washing with soap and water over using hand gel in standard precaution wards, limiting the number of team members entering patient rooms and reducing nursing procedures that require PPE, such as measuring urine output.

To protect staff, PPE training sessions must be updated and available to all the users.

It is recommended to adopt a policy limiting the number of visitors in hospitalised patient departments in order to protect both the public and patients from exposure and reduce the use of PPE, with rare exceptions, such as end-of-life circumstances. These decisions, taken together with patients, are difficult both for patients and families as well as medical personnel, who acknowledge the significant contribution of friend and family support for hospitalised cancer patients.

### Well-being of the healthcare team

Finally, the emotional and physical well-being of our staff requires proactive attention. Provider burnout is expected; therefore, priority must be given to protecting the health of frontline staff and ensuring a safe workplace environment. Furthermore, human resources should be attended to by developing policies and compensation for leaves of absence and mandatory isolation and creating work support groups. Reassignment from clinical tasks to administrative functions must be considered for immunocompromised staff or those having significant comorbidities that put them at higher risk of COVID19.

### Guidelines for healthcare staff [29, 30]

- Schedule and take short breaks from work to de-stress and attend to your basic needs. A few minutes of rest during your shift can be enough. Even a 5-minute walk can improve your energy and mental focus.
- Take time each day to do something that brings you joy, even if it is for a brief moment.
- Maintain a healthy diet; bring your own food to work.
- Keep your daily activities schedule as regular as you can.
- Take some sunlight.
- Try to practice chair yoga or stretch at work.
- Exercise regularly. Try to walk or bike to work if you can.
- Avoid or limit alcohol and caffeine consumption.
- Manage excessive fatigue, irritability, lack of concentration or anxiety.
- Take a moment to breathe slowly before entering a work area or entering or exiting a patient room. This can be difficult while you are wearing personal protective equipment, such as a mask, but breathing is relaxing and helps your body ease the physical symptoms of stress. If you consult regularly with a mental health professional, schedule video appointments or a telephone call. If you do not consult regularly with a mental health professional but do feel that it could be useful at this moment, schedule an appointment or call.
- If a spiritual practice is or has been important to you in the past, resume it.

### Outlook

The COVID-19 pandemic has posed unique challenges and learning opportunities for oncology centres. The future trajectory of this pandemic is uncertain and we must continue to prepare for its widespread impact. The situation is dynamic and policies and recommendations may change at any point. While we write this document, the health crisis surrounding COVID-19 continues to evolve, and circumstances may change some of our current recommendations.

We hope to overcome these uncertain times, even though the long-term economic and emotional impact will be two great future challenges. However, the overall objective is to continue to provide compassionate and safe care to cancer patients. We hope to continue to support our patients and the cancer community while this pandemic continues to spread globally.

## Consensus panel

### Questions for the panel

See Appendix II for questions formulated.

### Results

The panel was comprised of the following scientific societies: Argentine Society of Radiation Therapy (SATRO), Argentine Society of Cancerology (SAC), Argentine Society of Clinical Oncology (AAOC), Argentine Society of Surgery (AAC), Rosario Association of Oncology, Cancerology Society of La Plata, Tucuman Society of Clinical Oncology, Foundation of Oncologists of Argentine Patagonia (FOPA), Association of Oncologists of Cordoba, Society of Clinical Oncology of Chaco, Society of Clinical Oncology of Corrientes, Oncology Association of Formosa, Digestive Endoscopists of Buenos Aires (ENDIBA), Argentine Society of Gastroenterology (SAGE), CABA Oncology Network, Argentine Society of Mastology (SAM), AAMYCP and the Argentine Society of Urology (SAU).

The questions were presented to the panel and then distributed to the associates of the different scientific societies, whose responses were anonymous and not identified by name. This constituted a component for the face-to-face discussion, where results of the literature review were presented (sent in due course along with the first Delphi round), the survey open to medical professionals who treat oncology patients and the first round of the Delphi panel.

It is to be noted that the professionals, when responding to the open survey, did not have the document with the analysis and synthesis of the literature review prepared by the revising group, which reflected their usual practice. The analysis of 114 responses concluded that: regarding the question on triage or patient selection for first-time consultations or face-to-face consultations at their centres, there was a satisfactory implementation in 82% of cases. When asked about an established algorithm at their centres for patients with fever and suspicion of COVID-19, there was an affirmative response in 90% of cases.

For the question about when cancer patients who had finished their specific treatments would be considered at risk of developing severe forms of COVID-19, little over half (55%) responded only in those cases less than 6 months from completion of onco-specific treatment.

However, 34% did not take this variable into account and considered all those who had received treatment to be at risk. In relation to the question about adjuvant treatment in patients at risk of developing severe forms of COVID-19, 66% revealed inconsistent practices. For example, 33% decided to delay adjuvant treatment, while the remaining 33% decided to conduct curative treatment regardless of the COVID-19 situation. On the other hand, 18% decided to delay treatment in all cases. Different comments about this subject were sent, the majority of which agreed on the decision or individualisation of treatment by taking into consideration the risk-benefit ratio, consensus with the patient and also discussions within the interdisciplinary framework. For those deciding to delay treatment, a 60-day cut-off point was mentioned (two comments). One comment refers to the extreme situation, that is to say the catastrophe, under which it would also depend on the resources of the healthcare system and prioritisation of care. When asked about the use of radiation therapy and prioritising cases, 50% responded by referring to symptomatic advanced illness or oncological emergencies, preferably with hypofractionated schedules. Almost one third of cases (27%) included invasive cancer with curative intent, provided there was no neoadjuvant treatment option. 18% took no position in relation to radiation therapy. Regarding performing elective operations for cancer and the practice adopted in their medical society or care centre, 45% would not consider delaying surgery, since it would not be elective as it pertained to a serious disease such as cancer. However, one third of cases (26%) envisage postponing all surgical operations, and 23% consider that postponement should only occur in the context of containment and mitigation due to SARS-CoV-2. Responses to this question mention discussing postponement of certain types of surgery on a case-by-case basis, highlighting the importance of not missing the therapeutic window of opportunity—such as in the case of patients receiving neoadjuvant therapy. As a follow-up to the previous question, respondents were asked about the resection of metastases for remedial purposes: 45% answered that the decision was to proceed with surgery anyway to avoid missing the window of opportunity. Nearly half of the cases (43%) favour maintaining systemic treatment and rescheduling surgery. As regards elective surgeries and the health centre’s adoption of COVID-19 screening measures for patients in the at-risk category, in 79% of cases these are conducted through anamnesis and historical exposure records. In 15% of instances, it is mentioned that all preoperative cases undergo pharyngeal swabbing. In considering the treatment and strategy for cancer patients, 46% agree that treatment be decided in all cases following discussion by the multidisciplinary team, whereas 35% are of the opinion that a discussion should only be held in cases where this is warranted at the discretion of the treating physician.

Concerning the highly sensitive and important matter of PPE, or personal protective equipment, on this subject 64% of respondents agree or strongly agree with measures taken by their centre. Approximately, one third disagree or strongly disagree with measures taken or implemented by their centre.

A panel comprising the 17 scientific organisations met to discuss via video conference on 8 April, 2020.

Global and national epidemiological data were presented, and the definition of risk groups was shared, along with what has been published by the Ministry of Health on the handling of chronic noncommunicable diseases. Review of the document’s structure: based on evidence arising from three clinical practice guides (NCCN, ASCO and ESMO). An account was made of the methodology used: search, modified Delphi consensus. The result of the open-ended and non-standardised survey submitted to the participating organisations’ 114 professionals was presented in statistical format. The panel’s composition was outlined.

### Summary of outcomes for each of the questions discussed

**Question 1**: implementation of a triage system for oncological consultations: 80% agreement in the open survey, in agreement with the panel.

**Question 2**: organisational algorithm in response to the admissions circuit for patients with suspected or confirmed COVID-19 symptoms: agreement in both surveys.

Discussion: Chemotherapy clinics or oncology day units at hospitals are high-transit areas presenting a higher risk of contagion in the context of the COVID-19 pandemic. Patients (actively undergoing treatment and at greater risk of immunosuppression), accompanying persons and health care team members all transit through these areas. Implementing safety measures is a priority:

Implement social distancing in waiting rooms: restrict the number of accompanying persons to one per patient, adhere strictly to the schedule of appointments, establish spacious waiting areas or allow patients to walk around until their treatment begins (e.g., suggest that they leave the waiting room and call them on their phone once it is their turn for treatment).

Reduce daily patient admissions capacity: to avoid overcrowding in the waiting room and treatment room, assess the feasibility of increasing available days and extending time slots for treatment in order to curb the daily volume of patient admissions.

Implement a pre-entry triage: looking for symptoms indicative of a COVID-19 infection, carry out or reinforce its implementation if conducted prior to admission to the facility.

Respect social distancing in the treatment room: configure seats to ensure appropriate distance between patients and accompanying persons, and provide health care practitioners with sufficient room for manoeuvre.

Conduct methodical cleaning between patients: to this end we rely on the inter-institutional recommendations for preventing COVID-19, version 22/03/2020 SADI/SATI/ADECI/INE [16].

Implement appropriate handling and isolation measures in suspected cases.

**Question 3**: in relation to the time interval following the end of treatment taken as a risk variable:

Open survey: the majority of respondents indicated less than six months. Panel: 50% indicated less than six months. Not time-dependent 30%.

Discussion: Consideration given to assessing patients in line with prescribed monitoring controls. Three months and six months following the completion of treatment.

Even within the 12 month time interval, a stricter approach should be adopted towards the oncological monitoring plan. There is uncertainty regarding immunological recovery, which in some patients can be lengthy (up to 12 months), and should therefore be monitored.

The Wuhan case study report reveals that oncology patients stand a higher risk of suffering serious complications (50% compared to 16% in cancer-free patients, *p* = 0.0008) regardless of the treatment- or illness-free time interval. After adjusting to account for other variables such as age, smoking habits or other comorbidities, the risk was markedly higher (OR 5.34 IC 95% 1.80–16.18) in the subgroup where chemotherapy treatment had been completed or an operation performed in the preceding month. Consequently, the panel recommends that risk be discussed on a case-by-case basis in order to establish monitoring needs.

**Question 4**: adjuvant treatment in at-risk segment:

In an open survey, the majority chose to delay the start of treatment, contrasting with the panel’s strongly supported choice to follow standard procedure.

Discussion: Possibility of case-by-case discussion linked to the current phase of the pandemic in the country. Decisions need to be taken in the context of the pandemic’s evolving epidemiological landscape, with a particular focus on rationalising resources.

In light of the existing impact of the pandemic on comorbidity-free patients, the panel agrees to recommend that standard oncological treatment may be continued. For patients that are elderly and/or have comorbidities, assessments will be performed using the same criteria as under non-pandemic conditions.

The treatment’s absolute benefit and potential risks need to be weighed up for each pathology, targeting prioritisation and decision making within a multidisciplinary team context.

**Question 5**: therapeutic radiology, prioritisation

In an open survey, 50% pointed to limiting radiation therapy to urgent cases only, preferably through hypofractionation schemes. The panel is in agreement. 30% have not adopted a formal position on the matter.

Discussion: Certain definitive treatment modalities should be incorporated such as treatment for cancer of the cervix, lung, anus and adjuvant treatment of breast, head and neck cancer. Hypofractionated treatment modalities can be envisaged whenever feasible.

The timely and proper delivery of radiation therapy is a priority where some of the pathologies mentioned are concerned, in instances where hypofractionation (anal, cervical, and head and neck cancers) and concurrent radiotherapy and chemotherapy treatment modalities are not an option.

Tumours requiring prioritisation of concurrent radiotherapy and chemotherapy (RT-CT):

- **Cervical tumours:** definitive treatments using RT-CT. Prioritise brachytherapy. FIGO scale stage IV, discuss in committee the use of definitive versus hypofractionated palliative treatment.
- **Node-positive breast cancer:** consider hypofractionated treatment scheme over 3 weeks. Neoadjuvant therapies: reserve use for inflammatory carcinomas (the rest was explained in consensus).
- **Lymphomas:** prioritise in accordance with discussions held under committee for stages I and II of Hodgkin lymphoma and non-Hodgkin lymphoma.
- **Gastrointestinal tumours:** prioritise concurrent RT-CT treatment for locally advanced anorectal and oesophageal tumours. In the case of rectal tumours, assess treatment in five fractions.
- **Lung tumours:** stereotactic body radiotherapy for stages I or II. Hypofractionated concurrent RT-CT treatment delivered in 20 fractions for locally advanced tumours.
- **Head and neck:** apply standard practice for RT-CT.
- **Brain tumours such as glioblastoma multiforme:** patients with Recursive Partitioning Analysis III, under the age of 60, adapted extent of resection and reasonable functional state: hypofractionated treatment delivered in 15 fractions with temozolomide.

For such pathologies priority should be given to standard fractionation treatments, following prior multidisciplinary committee discussion.

**Question 6**: elective surgeries:

In the open survey referring to non-postponement because of the non-elective nature of surgeries, the panel’s view is to delay only in the containment stage and not to postpone.

Discussion: It must be considered within the context of the current phase of the pandemic, assessing the risk for complications on a case-by-case basis and the need for ICU. This extends to human resources, in terms of health care teams and treatment centre resources that are faced with the pandemic’s potential to evolve into a catastrophic situation. The natural progression of the illness also needs to be considered (e.g., pancreatic cancer where delaying would not be feasible), provided surgical interventions are curative in scope.

Prioritising surgeries in pathologies with curative criteria, otherwise refer the patient to concurrent therapies or chemotherapy. Multidisciplinary teams should discuss each case individually.

Furthermore, considering context: the progression of the pandemic compared to available resources in each centre.

Surgeons need to decide if they should switch surgical methods, from laparoscopic to open surgery. For COVID-19-positive patients, avoiding laparoscopic surgery or enhancing PPE use is recommended: for example, avoiding instruments that promote aerosolisation (such as the Harmonic scalpel) and reducing surgical teams to the minimum number of people. Temporarily suspending training and teaching during surgery is also recommended

**Question 7**: Resection of metastasis with curative criteria

Open survey, most respondents favour delaying surgery. The panel said surgery should still proceed.

The aim is to prioritise surgeries for patients who risk missing a therapeutic window (i.e., colorectal cancer patients with metastases after conversion treatment) based on an epidemiological assessment of COVID-19

**Question 8**: Elective surgery and screening patients to detect COVID-19:

Open survey, the panel agreed on only screening using clinical criteria.

Discussion:

What happens with asymptomatic patients in this community transmission phase?

Experience and studies from Wuhan show that major surgery for COVID-19-positive patients increases mortality.

Should clinical triage include swabbing?

Studies by Shaoqing *et al*. [31] and other authors [32] found that patients having major surgery should be swabbed as SARS-CoV-2 infection increases mortality. Infection also negatively impacts patients at high risk due to age and/or comorbidity as well as underlying oncological disease. These patients need chemotherapy, radiotherapy, or therapies with a high risk of neutropenia or immunosuppression.

**Question 9**: Multidisciplinary team discussion: the panel agreed to recommend multidisciplinary team discussion of all cases.

**Question 10**: PPE: There is a consensus that the measures adopted are adequate for institutions. Please consider that these recommendations may change.

Protecting patients must be a priority.

### Ethical considerations

Advance decisions and support at the end of life. Palliative care association document (AAMYCP).

Decisions amid scarce resources, such as ventilation criteria. There are algorithms for setting clinical and ethical decision making criteria.

Isolation at the end of life, crafting communication strategies to promote support.

There are common elements in all multidisciplinary discussions that have addressed distinct questions.

Decisions and recommendations should consider the national epidemiological situation (if it is not catastrophic).

Measures and their implementation must be considered based on resources, available medical equipment, and care centres in which each professional works.

The ideal framework for supporting medical decisions is a multidisciplinary discussion including all involved specialists and relies on the recommendations of scientific organisations.

## Final conclusions

A general recommendation is to provide adequate care to oncology patients in exceptional pandemic circumstances without missing opportunities to treat or cure them. Clinicians must also continue to provide professional support for all patients. This recommendation requires health professionals to strive to create multidisciplinary teams that can discuss the best, most timely measures for each situation.

Discussions continue about how to classify patients at greatest risk of developing severe forms of COVID-19, for example, patients who will have major surgery, chemotherapy or radiotherapy.

Is swabbing all patients before these therapies to detect asymptomatic infection adequate? If swabbing is feasible and is implemented systematically, how will it impact patients whom testing identifies as presymptomatic?

Another unresolved response issue is what some authors call the third wave: measuring the medium-term impact the pandemic has on response measures. For example, the consequences of delaying or interrupting care for chronic, non-communicable diseases, including cancer.

The relevant international scientific evidence is still under investigation. Incomplete evidence combined with constantly changing epidemiological circumstances poses unique challenges for treating cancer during a global pandemic. It is clear that health systems need organisational frameworks, such as preparation guidelines for all staff. The flexibility to adapt to change and a shared, multidisciplinary vision based on consensus are also vital. Institutions need these elements to continue treating cancer patients adequately amid uncertainty and rapid change.

## Conflicts of interest

The declaration of conflicts of interest was made by all members of the development group and participants in this consensus.

## Funding declaration

The group of experts states that no sponsorship was received for the development of the document.

## Disclaimer

This document represents the position of the participating Scientific Societies in the development of this recommendation. The recommendations are the product of a careful evaluation of the available evidence. In no case does it replace the clinical judgment of the treating physician, nor does it substitute for medical judgment in making appropriate decisions for each individual case, the consultation of the patient, the family or the caregivers.

## Appendix I: Organisations consulted, chair and/or designated representative

**Table:**

Organisations consulted, chair and/or designated representative			
	Institution	Acronym	President/Representative
1	Asociación Argentina de Oncología Clinica	AAOC	Santiago Bella, José María Lastiri
2	Sociedad Argentina de Terapia Radiante	SATRO	Gustavo Ferraris
3	Sociedad Argentina de Cancerología	SAC	Jorge Puyol, Darío Niewiadomski
4	Asociación Argentina de Cirugía	AAC	Sergio Quildrian, Pablo Sanchez
5	Asociación de Oncología de Rosario	AOR	Alejandro Chinellato
6	Sociedad de Cancerología de La Plata		Paola Price
7	Sociedad de Oncología Clinica de Tucuman		Luis Medina
8	Asociación de Oncólogos de Cordoba		Alejandro Ahualli
9	Endoscopistas Digestivos de Buenos Aires	ENDIBA	Rafael Escobar
10	Sociedad Argentina de Gastroenterología	SAGE	Edgardo Smecuol, Fabio Nachman
11	Sociedad Argentina de Mastología	SAM	Eduardo González
12	Asociación Oncología Clínica de Chaco		Guillermo Dellamea
13	Asociación Oncología Clínica de Corrientes		Julio Vélez
14	Asociación Oncología Clínica de Formosa		Silvana Rompato
15	Sociedad Argentina de Urología	SAU	Norberto Raúl Lafos, Juan Ramos Suppicich
16	Asociación Argentina de Medicina y Cuidados Paliativos	AAMYCP	Isabel Pincemin
17	Red de Oncología de CABA		Cristina Rosales, Claudia Bagnes
18	Fundación Oncológica de la Patagonia	FOPA	Ariel Zwenger

## Appendix II: Questions for panellists

1. Have you implemented a triage system for initial consultations or in-person appointments at your centre?
  - Yes
  - No
  - This clinical situation was not discussed
2. Your centre implemented an algorithm for appointments with patients who have a fever and suspected COVID-19
  - Ye
  - No
  - I do not know
3. How long do you believe cancer patients who have finished specific treatment have a higher risk of contracting severe forms of the COVID-19 disease?
  - Less than 6 months
  - Between 7 and 12 months
  - More than 12 months
  - Risk is not time-dependent
4. In patients who had a surgery, with curative criteria and risk factors for COVID-19 (due to comorbidity or age) your view of adjuvant treatment is to
  - Delay the start of treatment in all cases
  - Delay the start of treatment only if it includes chemotherapy
  - Follow normal practice standards despite the COVID-19 situation
5. In cases requiring the use of radiotherapy in cancer patients, you set priorities in the following cases
  - Infiltrating cancer with curative criteria as long as we have a neoadjuvant treatment option
  - Only for patients who have started radiotherapy treatment
  - In patients with advanced, symptomatic disease, but with hyperfractionation regimens
  - I have no specific opinion
6. Your centre/scientific organisation has taken the following position on performing elective surgery for cancer
  - Delay all elective surgeries
  - Delay surgeries only during the containment and mortality mitigation phase for COVID-19
  - Do not delay surgeries for cancer, they are not considered elective
7. For patients with advanced disease and the possibility of metastasis resection with curative criteria or intent, your decision is
  - Delaying surgery in virus transmission risk stage
  - Performing the surgery anyway, to avoid missing the window of opportunity
  - Continuing systemic treatment and reschedule surgery
8. For patients with scheduled elective surgery, your centre has adopted the following measures for screening patients (for COVID-19)
  - Do pharyngeal swabs for COVID-19 and chest CT scans for all patients
  - Do pharyngeal test / swab in all preoperative cases
  - Only screen using medical and exposure histories
9. In this situation, in the context of the SARS-CoV-2 pandemic, for all cancer patients, define strategy based on:
  - Multidisciplinary discussion in all cases
  - Only in cases that can be explained, based on the criteria of the treating medical doctor
  - Only in cases that have a unique, case-specific question
10. Are the measures your centre has taken to manage personal protective equipment (PPE) appropriate? Indicate the option that matches your opinion best
  - Strongly agree
  - Agree
  - Agree somewhat
  - Do not agree at all

## Appendix III: National scientific organisation guidelines included in the document

- Sociedad Argentina de Terapia Radiante (SATRO): http://www.satro-radioterapia.com.ar
- Sociedad Argentina de Cancerología (SAC): http://www.socargcancer.org.ar/2020_COVI-19.php
- Asociación Argentina de Oncología Clinica (AAOC): http://www.aaoc.org.ar/#/actividades-cientificas/1340/coronavirus-recomendaciones-para-pacientes-oncologicos
- Asociación de Oncología de Rosario: https://asoconcorosario.wixsite.com/website?fbclid=IwAR3MPH0b_7MTQ_oWwyVetbu3oLvishNr2BYhrN71xlP0bSqj_3tb8wW26Ck
- Sociedad de Cancerología de La Plata: https://www.facebook.com/SociedaddeCancerologiaLaPlata/
- Sociedad de Oncología Clinica de Tucuman: https://www.colemed.com/index.php/sociedades
- Asociación de Oncólogos de Cordoba: https://www.aocc.org.ar/autoridades/
- Endoscopistas Digestivos de Buenos Aires (ENDIBA): https://www.endiba.org.ar/coronavirus.php
- Sociedad Argentina de Gastroenterología (SAGE): https://sage.org.ar/wp-content/uploads/2020/03/Actualizacio%CC%81n-posicion-amiento-GADECCU.pdf.pdf.pdf
- Sociedad Argentina de Mastología (SAM): https://www.samas.org.ar/
- Asociación Argentina de Medicina y Cuidados Paliativos (aamycp): https://aamycp.com.ar/covid19/
- Sociedad Argentina de Cirugía: http://aac.org.ar/newsletter/2020/comunicado_23-3.htm
